# Characterization of the Interaction Between the Small Regulatory Peptide SgrT and the EIICB^Glc^ of the Glucose-Phosphotransferase System of *E. coli* K-12

**DOI:** 10.3390/metabo2040756

**Published:** 2012-10-16

**Authors:** Anne Kosfeld, Knut Jahreis

**Affiliations:** 1 Centre for Pathology and Forensic and Genetic Medicine, Institute for Human Genetics-Hannover Medical School, Carl-Neuberg-Str.1, D-30625 Hannover, Germany; 2 Department of Biology and Chemistry, University of Osnabrück, Barbarastr.11, D-49069 Osnabrück , Germany

**Keywords:** glucose transporter, glucose signaling, PTS, regulation, protein–protein interaction, SgrT, *E. coli* K-12

## Abstract

*Escherichia coli* is a widely used microorganism in biotechnological processes. An obvious goal for current scientific and technical research in this field is the search for new tools to optimize productivity. Usually glucose is the preferred carbon source in biotechnological applications. In *E. coli*, glucose is taken up by the phospho*enol*pyruvate-dependent glucose phosphotransferase system (PTS). The regulation of the *ptsG* gene for the glucose transporter is very complex and involves several regulatory proteins. Recently, a novel posttranscriptional regulation system has been identified which consists of a small regulatory RNA SgrS and a small regulatory polypeptide called SgrT. During the accumulation of glucose-6-phosphate or fructose-6-phosphate, SgrS is involved in downregulation of *ptsG* mRNA stability, whereas SgrT inhibits glucose transport activity by a yet unknown mechanism. The function of SgrS has been studied intensively. In contrast, the knowledge about the function of SgrT is still limited. Therefore, in this paper, we focused our interest on the regulation of glucose transport activity by SgrT. We identified the SgrT target sequence within the glucose transporter and characterized the interaction in great detail. Finally, we suggest a novel experimental approach to regulate artificially carbohydrate uptake in *E. coli* to minimize metabolic overflow in biotechnological applications.

## 1. Introduction

With its fast growth and simple cultivation *Escherichia coli* is a widely used microorganism in biotechnological processes and in industrial microbiology. One of the most important applications of recombinant DNA technology is the genetic manipulation of *E. coli* K-12 for the production of human insulin [1]. Modified *E. coli* strains are also currently used for the synthesis of different enzymes, amino acids and other peptide hormones. To maximize productivity, *i.e.*, the yield in relation to duration and costs, it is essential to permanently optimize the biotechnological process. One major problem during high density growth of *E. coli* K-12 is the production and excretion of acetate, which affects growth and recombinant protein expression [2,3]. To circumvent this, different growth strategies [3] have been applied as well as targeted changes in central carbon metabolism [2,4] or control of the glucose transport process has been modified [5,6]. Especially the latter approach seems to be very helpful since acetate excretion mainly occurs when the transport rate exceeds the metabolism which causes a temporal metabolic imbalance. 

In *E. coli*, glucose is taken up by the phospho*enol*pyruvate (PEP)-dependent glucose-phosphotransferase-system (Glc-PTS) [7]. Phosphotransferase systems usually consist of two cytoplasmic energy-coupling proteins, Enzyme I (EI, gene *ptsI*) and Histidine-containing protein (HPr, gene *ptsH*), and in particular for *E. coli* K-12 of a range of more than 20 different carbohydrate spe­cific Enzymes II (EIIs), which catalyze concomitant carbohydrate transport and phosphorylation [8]. The first step in the PTS-typical phosphorylation-chain is catalyzed by EI, a PEP-dependent protein-kinase. The use of PEP, an intermediate of glycolysis, as a phosphoryl group donor couples tightly carbohydrate transport and metabolism. In the case of the Glc-PTS, the phosphate group is subsequently transferred from EI~P to HPr, from HPr~P to the soluble EIIA^Glc^ (sometimes also called EIIA^Crr^, gene *crr*), and finally from EIIA^Glc^~P to the glucose-specific membrane protein EIICB^Glc^ (gene *ptsG*), which is responsible for glucose uptake and phosphorylation. EIICB^Glc^ (50.7 kDa) consists of two functional domains, the membrane bound EIIC^Glc^ domain (41.1 kDa) and the cytosolic EIIB^Glc^ domain (9.6 kDa). The EIIC^Glc^ domain forms a stable homodimer in the membrane and is responsible for glucose uptake, whereas the EIIB^Glc^ is located in the cytoplasm and phosphorylates the glucose [9]. Both domains are connected through a flexible linker. The linker is surface exposed, since a proteolytic cleavage within the linker is possible [10]. Phosphorylation of EIICB^Glc^ protects against protease cleavage, suggesting a conformational change of this region during glucose uptake [10]. The linker shows the highly conserved amino acid sequence KTPGRED (aa 382-388) which is present in most of the PTS transport proteins of the glucose/N-acetyl-glucosamine family. The function of this motif was unclear so far [7]. This motif appears to be nonessential for transport, since alanine substitutions show no or only a slight effect with the exception of EIICB^Glc^G385A which exhibited a highly reduced phosphorylation activity of less than 10% of wild type activity [10,11]. Only a complete deletion of this sequence led to a total loss of transport and phosphorylation activity [12].

Regulation of the *ptsG* gene for the EIICB^Glc^ is very complex and occurs both at the levels of transcription and posttranscriptional control. The major specific regulator of *ptsG* expression is the repressor Mlc (mnemonic for makes large colonies, previously DgsA, gene *dgsA*), which is inactivated by glucose in the medium. In contrast to other repressors, induction of Mlc is not catalyzed by direct binding of glucose, or by any other small molecular inducer. Instead, as part of an unusual regulatory mechanism, the membrane-bound EIICB^Glc^ binds Mlc, but only when it is in its dephosphorylated form. Thus, in the absence of glucose, Mlc binds to its target promoter/operator *ptsGop*, while in the presence of glucose, the dephosphorylated EIICB^Glc^ sequesters the repressor away from its operator, allowing enhanced *ptsG* transcription [13,14,15,16]. Besides this main regulation via the glucose repressor Mlc, several other global factors were identified. These are cAMP-CAP [17], ArcA [18], SoxS [19], Fis [20] and two alternating sigma factors σ^32^ [21] and σ^S^ [22]. In addition to these transcriptional regulation mechanisms, a posttranscriptional regulation system, the so-called *sgrRST*-system [23,24], was identified as regulating *ptsG* mRNA stability as well as transport activity of EIICB^Glc^. Accumulation of glucose-6-phosphate (Glc6-P) or fructose-6-phosphate (Fru6-P) in the cell activates the transcriptional activator SgrR which, in turn, is responsible for the activation of the small regulatory sRNA SgrS [24]. SgrS itself has two functions. On the one hand, it is capable of forming Hfq-dependent RNA-RNA hybrids with the *ptsG* mRNA, a first step in RNaseE dependent specific degradation of this mRNA. On the other hand, the *sgrS* gene encodes a small protein of 43 amino acids called SgrT which is responsible for downregulation of glucose transport [25].

The recent discoveries of these two posttranscriptional regulatory mechanisms provide new approaches to control glucose uptake under various growth conditions. For example Negrete *et al.* managed to reduce acetate excretion of glucose fermenting *E. coli* cells by overexpressing SgrS [26]. Likewise, exclusive overproduction of SgrT led to a drastic reduction of cell growth in minimal medium with glucose as a sole carbon source [25]. This gave the first hint that the Glc-PTS might be a direct target of SgrT and that the functions of SgrS and SgrT are redundant. Subsequently, Gabor *et al.* [27] repeated this growth experiment using minimal medium with sucrose as a single carbon source. For this experiment, an *E. coli* derivative was used, which shares all the components of the typical PTS cascade with the Glc-PTS (EI, HPr, EIIA^Glc^) with the exception of the sucrose specific EIIBC^Scr ^ [28]. This transporter protein like the EIICB^Glc^ belongs to the glucose/N-acetyl-glucosamine–sucrose/ß-glucosides superfamily of EII proteins [29]. However, the sucrose specific transporter has a different order of the two functional domains and lacks a conserved KTPGRED motif in the linker region between these two sites. Overproduction of SgrT did not interfere with cell growth in minimal medium with sucrose providing a first hint that indeed EIICB^Glc^ and no other component of the Glc-PTS might be the SgrT target [27].

In this study we focused our interest on the regulation of EIICB^Glc^ activity by SgrT. We identified the SgrT target sequence within EIICB^Glc^ and characterized the interaction between the glucose transporter and the small regulatory peptide in great detail. This may eventually lead to novel approaches to minimize metabolic overflow and thus improve the feasibility of the use of *E. coli* in biotechnological applications.

## 2. Results and Discussion

### 2.1. SgrT Binds to Dephosphorylated EIICB^Glc^ in *in vivo* Crosslinking assays

**Figure 1:**
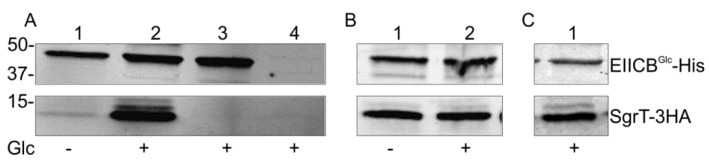
Crosslinking experiments with EIICB^Glc^ and SgrT in different genetic backgrounds.

In order to test the assumption of a direct protein–protein interaction between SgrT and EIICB^Glc^, we performed an *in vivo* crosslinking experiment with paraformaldehyde. With this method, even weak *in vivo* interactions between two proteins are detectable in the case that the proteins are in close proximity to each other (2 Å or less) [30]. To identify the two interaction partners in subsequent Western blots, both proteins were tagged with different flags (EIICB^Glc^-5His, SgrT-3HA). Both tagged proteins were fully functional in complementation assays, e.g., glucose transport in a *ptsG* deletion background in the case of the EIICB^Glc^-5His protein or reduction of growth in minimal medium with glucose as a sole carbon source in the case of the SgrT-3HA peptide [27]. The two proteins were expressed in a double deletion strain (JKA12) and the cells were treated with paraformaldehyde. The cells were disrupted by sonification, membrane proteins were solubilized and EIICB^Glc^-His and proteins binding to it were purified with Ni-NTA agarose. The resulting Western blot analysis showed a strong copurification of SgrT and thus an interaction of SgrT and EIICB^Glc^ in the presence of glucose in the medium (Figure 1A, lane 2). Interestingly, only a very weak interaction could be detected in cells grown in the absence of glucose (Figure 1A, lane 1). No signals for SgrT-3HA were obtained in a *sgrT*HA deletion background (Figure 1A, lane 3) or in a *sgrT*HA^+^/ *ptsG*His (Figure 1A, lane 4) deletion strain. The latter result demonstrates that the detection of SgrT-3HA clearly depends on the presence of EIICB^Glc^.

(**a**) Lanes 1 and 2 show crosslinking experiments with strain JKA12 (LJ110*ΔptsG::cat ΔsgrRST::neo*) transformed with two plasmids expressing EIICB^Glc^-His (pRR48GH) and SgrT-3HA (pACYC184sgrT3HA). Cells were grown in the absence or presence of glucose as indicated; molecular weight markers are given on the left side (in kDa). The results show an interaction of SgrT and EIICB^Glc^ in the presence of glucose. Control experiments are illustrated in lane 3 (JKA12 transformed with pRR48GH and pACYC184) and lane 4 (JKA12 transformed with pRR48 and pACYC184sgrT3HA). In both cases, no signals for SgrT-3HA could be observed.
(**b**) Lanes 1 and 2 show crosslinking experiments with the *ptsHIcrr* deletion strain LJ140 transformed with pRR48GH and pACYC184sgrT3HA. Cells were grown in the absence or presence of glucose as indicated.
(**c**) Lane 1 shows a crosslinking experiment with the *dgsA* deletion strain LJB17 transformed with pRR48GH and pACYC184sgrT3HA. Cells were grown in the presence of glucose. This result indicates an Mlc-independent interaction between EIICB^Glc^ and SgrT.

Glucose uptake leads to a net dephosphorylation of EIICB^Glc^ and to conformational changes of the transporter during the uptake process. To test whether dephosphorylation and no glucose induced conformational change of the transporter is sufficient for SgrT binding, this experiment was repeated in a *ptsHIcrr* deletion strain (LJ140), where no phosphorylation of EIICB^Glc^ can occur. The results shown in Figure 1B indicate an interaction between SgrT and EIICB^Glc^ both in the presence and in the absence of glucose, indicating that SgrT binds to dephosphorylated EIICB^Glc^ with a much higher preference and that conformational changes of the EIICB^Glc^ induced by glucose transport are not involved in SgrT binding. Dephosphorylated EIICB^Glc^ also binds and sequesters the glucose repressor Mlc in the process of *ptsG* induction. To see whether SgrT binding to dephosphorylated EIICB^Glc^ depends on the presence of Mlc, we repeated the crosslinking experiment in an *mlc* (*dgsA*) deletion background. As shown in Figure 1C, an Mlc-independent interaction between EIICB^Glc^ and SgrT was detected. This finding supports the idea of a direct SgrT-EIICB^Glc^ interaction.

### 2.2. SgrT Binds to Full Length EIICB^Glc^ and to Its Truncated EIIC-Linker Derivative in Bimolecular Fluorescence Complementation Assays

The previous results showed that SgrT interacts with the unphosphorylated full-length EIICB^Glc^ in crosslinking assays. In order to narrow the region of the EIICB^Glc^ interaction side, we performed bimolecular fluorescence complementation assays [31] with different subdomains of the glucose transporter. In these assays, both proteins of interest are linked to one half of a green fluorescent protein (Gfp) protein. In case of interaction, both halves regenerate a fluorescent full-length protein.

**Figure 2:**
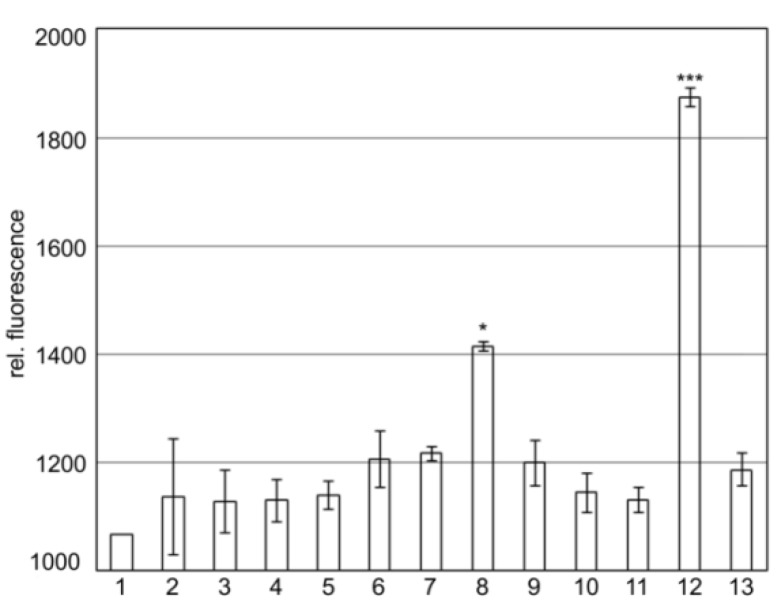
Bimolecular fluorescence complementation assays with different EIICB^Glc^ derivatives and SgrT.

Results shown in Figure 2 indicate that SgrT interacts with the full-length EIICB^Glc^ protein (Figure 2, lane 8) as well as with the EIIC^Glc^-linker domain without EIIB^Glc^ (Figure 2, lane 12). The interaction between SgrT and EIIC^Glc^-linker is even higher compared to the full length protein. This might indicate that a deletion of the EIIB^Glc^-domain exposes the linker, which thus becomes a better target for SgrT. In contrast, no interaction between SgrT and the EIIC^Glc^ domain without the linker could be observed (Figure 2, lane 11). Interestingly, there was also no interaction between the soluble EIIB^Glc^ with or without the linker domain and SgrT (Figure 2, lanes 9 and 10). This could be a hint that either the C-domain also plays at least some role in interaction or that a membrane environment is required for the interplay.

The relative fluorescence units were measured for different EIICB^Glc^ derivatives and SgrT both fused to one half of the green fluorescent protein to determine the amounts of bimolecular fluorescence complementations. Strain JKA17 (BL21(λDE3)*ΔptsG::cat*) was transformed with various plasmids expressing different Gfp-fusion proteins. Equal amounts of cells were used and each culture was inoculated and measured at least three times. For determination of background fluorescence, a leucin-zipper fused to the N- or C-terminal part of GFP was used as follows: Z-NGFP (pET11a-Z-NGFP) and Z-CGFP (pMRBAD-Z-CGFP). For description of plasmid construction and experimental procedure, see experimental section.

Results are given for the following sample combinations: 

1. Z-NGfp/EIICB^Glc^-CGfp; 2. SgrT-NGfp/Z-CGfp; 3. Z-NGfp/EIIB^Glc^-CGfp; 4. Z-NGfp/Linker-EIIB^Glc^-CGfp; 5. Z-NGfp/EIIC^Glc^-CGfp; 6. Z-NGfp/EIIC^Glc^-Linker-CGfp; 7. Z-NGfp/EIIC^Glc^-Linker-P384R-CGfp; 8. SgrT-NGfp/EIICB^Glc^CGfp; 9. SgrT-NGfp/EIIB^Glc^-CGfp; 10. SgrT-NGfp/Linker-EIIB^Glc^-CGfp; 11. SgrT-NGfp/EIIC^Glc^-CGfp; 12. SgrT-NGfp/EIIC^Glc^-Linker-CGfp; 13. SgrT-NGfp/EIIC^Glc^-Linker-P384R-CGfp. 

The results indicate that there is relative background fluorescence up to 1200 units in control cultures (lanes 1 to 7). The same relative fluorescence was detected for the combinations of SgrT-NGfp with EIIB^Glc^-CGfp (lane 9), Linker-EIIB^Glc^-CGfp (lane 10), EIIC^Glc^-CGfp (lane 11) and EIIC^Glc^-linker-P384R-CGfp (lane 13), meaning that there is no interaction between SgrT and these EIICB^Glc^-domains. A significantly higher relative fluorescence was detected between SgrT-NGfp and EIICB^Glc^-CGfp (lane 8) and EIIC^Glc^-linker-CGfp (lane 12), respectively. These results indicate an interaction between SgrT and the full-length protein EIICB^Glc^ or the Linker-EIIC^Glc^-domain, respectively. Significance levels: *p = 0.05, ***p = 0.001.

### 2.3. The KTPGRED Motif in the Linker Region of EIICB^Glc^ is the Main SgrT Target Sequence

In a previously published experiment, we identified the single amino acid substitution P384R in EIICB^Glc^, which caused a complete release of SgrT inhibition during growth in minimal medium with glucose as a sole carbon source [27]. The amino acid P384 is located within the conserved KTPGRED motif. The function of this region was unknown until now, but it seems to play an important role in SgrT regulation. Accordingly, as indicated in Figure 2, lane 13, no bimolecular fluorescence complementation was detected for SgrT-NGfp and EIIC^Glc^-linker-P384R-CGfp.

**Figure 3:**
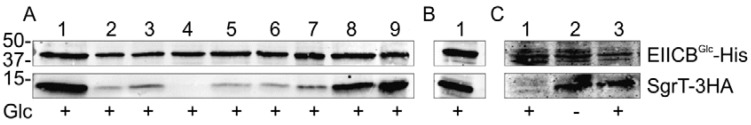
Crosslinking experiments with different KTPGRED mutants of EIICB^Glc^ and SgrT.

To identify other functionally important amino acid residues, we performed SgrT-EIICB^Glc^ crosslinking assays with single amino acid substitutions in the KTPGRED motif of the glucose transporter. All amino acid residues of this motif were replaced by the small and hydrophobic amino acid residue alanine. In addition, EIICB^Glc^ P384R was also reanalyzed in this test. The obtained EIICB^Glc^ derivatives were capable of complementing a *ptsG* deletion strain on a MacConkey glucose (McCGlc) plate, even EIICB^Glc^ G385A (data not shown). This indicates that under high glucose concentrations (1% in McC plates) the residual activity of all mutants is sufficient to complement transport activity and that all proteins are folded correctly. Similarly important is the fact that all proteins were stable and could be purified easily. Cells overexpressing SgrT and the respective EIICB^Glc^ derivative were grown in rich medium in the presence of glucose and treated with paraformaldehyde. Subsequently, cells were disrupted and EIICB^Glc^-His was purified with Ni-NTA agarose. Respective SgrT co-purifications were visualized by Western blot analysis. As shown in Figure 3A the strongest effect was exhibited by the P384R substitution, which completely abolished the interaction between the two proteins. Strong effects were also caused by the substitutions T383A, P384A, G385A, R386A and E387A. Compared to the wild type protein almost no effects were obtained for the substitutions K382A and D388A. This might indicate that the crucial residues for the EIICB^Glc^ - SgrT interaction are in the center of this sequence motif.

(**a**) This part of the figure shows crosslinking experiments with strain JKA12 (LJ110*ΔptsG::cat ΔsgrRST::neo*) transformed with two plasmids expressing SgrT-3HA (pACYC184sgrT3HA) and wild type EIICB^Glc^-His (lane 9) or different EIICB^Glc^-His-derivatives (lanes 1-8). Cells were grown in the presence of glucose as indicated; molecular weight markers are given on the left side (in kDa). The following EIICB^Glc^ derivatives were used in combination with SgrT-3HA:
  EIICB^Glc^-K382A-His; 2. EIICB^Glc^-T383A-His; 3. EIICB^Glc^-P384A-His; 4. EIICB^Glc^-P384R-His; 5. EIICB^Glc^-G385A-His; 6. EIICB^Glc^-R386A-His; 7. EIICB^Glc^-E387A-His; 8. EIICB^Glc^-D388A-His; 9. EIICB^Glc^-His (wild type). These results indicate that the crucial residues for the interaction between the two proteins are in the center of the KTPGRED motif.
(**b**) Lane 1 shows a crosslinking experiment with strain JKA12 expressing SgrT-3HA (pACYC184sgrT3HA) and the so called “relaxed” mutant EIICB^Glc^-V12F-His (pRR48GH-V12F). Cells were grown in the presence of glucose. These results indicate an interaction between SgrT and the “relaxed” derivative of EIICB^Glc^.
(**c**) This part of the figure shows crosslinking experiments between SgrT-3HA and the “locked in” mutant EIICB^Glc^-K150E-His (pRR48GH-K150E) in different genetic backgrounds. Lane 1 shows a sample of strain JKA12 expressing SgrT-3HA and EIICB^Glc^-K150E-His, lanes 2 and 3 exhibit samples of LJ140 expressing the same proteins. Cells were grown in the absence or presence of glucose as indicated. These results indicate no interaction between SgrT and EIICB^Glc^K150E in a PTS-positive strain, but a strong interaction in a *ptsHIcrr* deletion background.

The mutation P384R in EIICB^Glc^ has previously been described to cause a so-called “relaxed” conformation [16], which allows a facilitated transport of substrates like mannose, glucosamine or fructose. The exact nature of the conformational difference, however, is still unclear. To test whether this “relaxed” conformation in general interferes with the SgrT interaction, we tested the EIICB^Glc^V12F derivative in the crosslinking assay. This mutation has also been attributed with properties which cause the same “relaxed” phenotype and thus belongs to the same class of mutants [10]. As shown in Figure 3B, in contrast to EIICB^Glc^P384R the unphosphorylated V12F derivative exhibited a strong interaction with SgrT, which means that a “relaxed” conformation *per se* has no influence on the interplay with this small regulatory peptide.

A different class of mutations, such as those caused by the substitution K150E, leads to a so-called “locked-in” conformation of EIICB^Glc^. In this case, the transporter can be phosphorylated but cannot transfer the phosphate group to the bound glucose molecule [10]. Thus, the transporter remains phosphorylated and cells carrying this mutation are not capable of transporting glucose by the Glc-PTS. Accordingly, no interaction between SgrT and EIICB^Glc^K150E could be detected in PTS-positive strains in the presence of glucose (Figure 3C, lane 1). In contrast, the introduction of a *ptsHIcrr* deletion, however, as for the wild type protein, caused a constitutive interaction between SgrT and EIICB^Glc^K150E (Figure 3C, lanes 2 and 3) which again emphasizes the strong correlation between the phosphorylation level and the ability to interact with SgrT.

### 2.4. Recruitment of SgrT to the Membrane by EIICB^Glc^ Can Be Visualized By *in vivo* Fluorescence Microscopy

**Figure 4:**
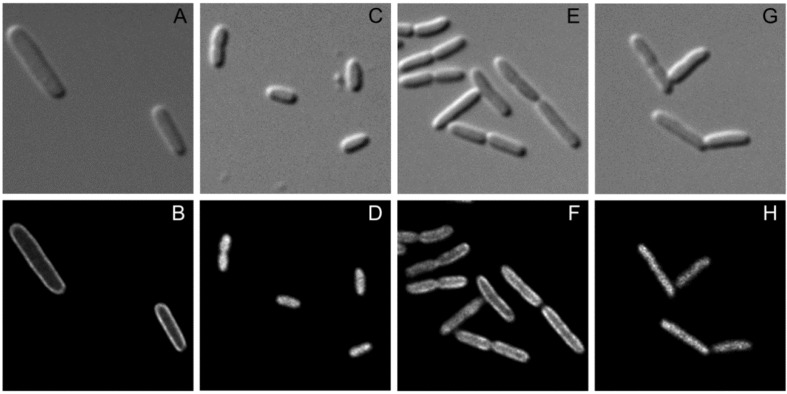
Fluorescence microscopy for the determination of EIICB^Glc^ and SgrT localization.

For further analysis of the interaction between SgrT and EIICB^Glc^ we performed fluorescence microscopy to find out more about the distribution pattern of the two proteins in living cells. As shown in Figure 4, plasmid encoded EIICB^Glc^ tagged with Gfp was homogeneously distributed in the cytoplasmic membrane (4B), whereas SgrT tagged with Gfp could be detected in an EIICB^Glc^-negative strain only in the cytosol (4D). In contrast, in *E. coli ptsG*^+^ cells that were grown in the presence of glucose, the localization of SgrT-Gfp clearly shifted to the membrane, which indicates a sequestration of SgrT by unphosphorylated EIICB^Glc^ (4F). In accordance with the previously obtained results of the crosslinking experiments, EIICB^Glc^P384R, unlike the wild type protein, was not capable of sequestering SgrT-Gfp (4H), which, yet again, indicates the missing interaction between the two proteins.

Bright field (upper lane) and fluorescence microscopy (lower lane) were performed with three different strains expressing EIICB^Glc^ or SgrT derivatives tagged with Gfp.

**A** and **B**: JKA12 (*ΔptsG::cat ΔsgrRST::neo*) expressing EIICB^Glc^-Gfp; **C** and **D**: JKA12 expressing SgrT-Gfp; **E** and **F**: JKA1 (*ptsG*^+^*ΔsgrRST::neo*) expressing SgrT-Gfp. **G** and **H**: JKA18 (*ptsG*_P384R_*ΔsgrRST::neo*) expressing EIICB^Glc^P384R and SgrT-Gfp. All cells were grown in minimal medium with 0.2% glucose. These results indicate a sequestration of SgrT by unphosphorylated EIICB^Glc^ (wild type), but not by EIICB^Glc^P384R in living cells.

### 2.5. Discussion

Overflow metabolism, which is accompanied in *E. coli* by acetate production, is a metabolic phenomenon which takes place when the rates of carbohydrate transport and glycolysis exceed a critical value due to high growth rates under aerobic growth conditions [32]. Acetate is produced from acetyl-CoA via acetyl-phosphate. Thus, under conditions of high glycolytic flux overflow metabolism directs a portion of the excess acetyl-CoA to acetate production. In addition, other byproducts such as succinate, lactate, pyruvate, or methylglyoxalate can also be produced under these conditions. During overflow metabolism, not all of the substrate is converted into biomass which constitutes an enormous disadvantage for biotechnological processes. Accordingly, the phenomenon of overflow metabolism has been investigated in greater depth during the past years in an effort to make industrial biotechnology more cost-efficient and economically advantageous [33].

The preferred carbon source in biotechnological applications is glucose, which in *E. coli* is usually taken up by the glucose-phosphotransferase system. The regulation of the *ptsG* gene encoding the glucose transporter EIICB^Glc^ is extremely complex. At least seven different proteins are involved in the transcriptional control (reviewed in [7]). Moreover, several research groups have identified and characterized the function of the *sgrRST*-system, which creates a sophisticated posttranscriptional feedback regulation mechanism of glucose transport during intracellular Glc6-P or Fru6-P accumulation [23,24,26,27,34,35]. Whereas the small regulatory RNA SgrS destabilizes *ptsG* m-RNA, the function of the small regulatory peptide SgrT, which is simultaneously encoded by the *sgrS* gene, has not been very well characterized thus far.

In this paper we could demonstrate for the first time by several experimental approaches, that the highly conserved KTPGRED motif in the linker region between the EIIC^Glc^ and the EIIB^Glc^ domains of the glucose transporter constitutes the SgrT target site. Furthermore, using site-directed mutagenesis, we were able to identify the most important residues for this protein–protein interaction. These findings finally provide a good explanation for the existence of this highly conserved motif within an otherwise non-conserved region of the protein. Moreover, we could demonstrate that according to the physiological needs, almost exclusively dephosphorylated EIICB^Glc^ interacts with SgrT. During glucose uptake EIICB^Glc^ conducts several conformational changes which result in glucose translocation and phosphorylation [36]. Glucose is bound with high affinity on the periplasmic site of the inner membrane. Subsequently, the protein conformation changes into an occluded state (the glucose is completely surrounded by the protein). Phosphorylation of the substrate again causes a conformational change and thus leads to a decreased affinity and to the release of Glc6-P into the cytoplasm [36]. Erni *et al.* could demonstrate that the flexible linker which connects the phosphorylated EIIB^Glc^-domain with the glucose binding EIIC^Glc^-domain conducts severe conformational changes during this transport process [10]. This provides an explanation for the clear differences observed in SgrT binding between phosphorylated and unphosphorylated EIICB^Glc^.

Furthermore, transport activity of EIICB^Glc^ is influenced by different amino acid substitutions which can be dissected into three groups: The first group consists of mutations which cause “relaxed” substrate specificity. These single amino acid substitutions are scattered over the entire EIIC^Glc^ domain and allow facilitated diffusion of substrates like mannose and glucosamine [16,37], fructose [38], ribose and xylose [39], mannitol [40], ribitol und arabinitol, respectively (reviewed in [10]). In contrast, so-called “uncoupled” mutants exhibit a separation of translocation and phosphorylation and the uptake of unphosphorylated glucose, for example, in a *ptsHICrr* deletion background [29,41]. The third group consists of so-called “locked-in” mutations that displayed a radical decrease in transport, but unchanged phosphorylation activity [42]. Our results indicate that none of these specific or intermediate conformations has an influence on the interaction with SgrT and that the biggest conformational differences seem to exist between the phosphorylated and the unphosphorylated EIICB^Glc^.

In spite of the SgrS/SgrT feedback regulation loop of glucose transport, *E. coli* tends to produce acetate during high cell density fermentation. However, the *sgrRST*-system provides new regulatory tools to artificially modify glucose uptake rates according to biotechnological needs. Negrete *et al.* already demonstrated that overexpression of SgrS is sufficient to reduce acetate excretion of glucose fermenting *E. coli* cells [26]. In addition, we and others have demonstrated that the exclusive overproduction of SgrT causes a drastic reduction of bacterial growth in minimal medium with glucose, but not with sucrose as sole carbon source [25,27]. In principle, it should be possible to couple the production of SgrT more strictly to the glycolytic flux, for example by isolating SgrR mutants with enhanced affinity to its molecular inducer. In this case, the slightest accumulation of these metabolites should result in a shutdown of glucose transport and should minimize the overflow. After identification of the SgrT target sequence it should be possible to incorporate this target box into other carbohydrate transport proteins to create an artificial control system in order to achieve the desired uptake rates. Especially sucrose may be a sought-after alternative for a cheap carbohydrate source, as sugar-cane molasses is available in great quantities.

## 3. Experimental Section

*Media and growth conditions.* Cells were grown routinely either in Luria broth without glucose and calcium ions (LB_0_), or in 2xTY medium as described [55]. Antibiotics were used at the following concentrations: tetracycline (Tc) 10 mg/L, kanamycin (Kn) 25 mg/L and ampicillin (Ap) 50 mg/L, respectively. Minimal medium supplemented with 0.2% carbon source was used as indicated [56]. IPTG was used at concentrations of 100µM-500µM for induction of protein production in growth inhibition assays and at a concentration of 1 mM for induction of protein production for crosslinking experiments. Cells were incubated at 37 °C with shaking.

*Bacteria strains and plasmids.* All strains used were *E.coli* K-12 derivates. Table 1 and Table 2 list the genotypes and sources of the relevant bacterial strains and plasmids. Oligonucleotides are listed in Table 3 in the supplemental materials. Alleles were moved between strains by P1-transduction (performed as described previously [47]) or inserted via λ Red recombination [44].

**Table 1:** Strains and Phages used in this study.

Strains	Relevant Genotype or Phenotype	Source or Reference
*Escherichia coli*		
BL21 (λDE3)	* fhuA2 [lon] ompT gal (λ DE3) [dcm] ΔhsdS λ DE3 = λ sBamHIo ΔEcoRI-B int::(lacI::PlacUV5::T7 gene1) i21 Δnin5 *	[43]
BW25113	*lacI^q^ rrnB_T14_ ΔlacZ_WJ16_ hsdR514 ΔaraBAD_AH33_ ΔrhaBAD_LD78_*	[44]
JKA1	LJ110 *ΔsgrRST::cat^+ ^*(Cam^R^)	this study
JKA12	LJ120 *ΔsgrRST:: neo^+ ^*(Kan^R^)	this study
JKA17	BL21 (λDE3) *ΔptsG::cat^+ ^*(Cam^R^)	this study
JKA18	LJB5 *ΔsgrRST:: neo^+ ^*(Kan^R^)	this study
JM109	*thi-1* *Δ(lac-proA,B)U169 gyrA1,96 recA1 endA1 relA1 hsdR17 supE44/F´traD36 proA^+^B^+^lacI^q^lacZ* *ΔM15*	[45]
K-12	Wildtyp	K. Jahreis, lab stock
LJ110	W3110 Fnr+	[16]
LJ120	LJ110 *ΔptsG::cat^+ ^*(Cam^R^)	[16]
LJ140	LJ110 *ΔptsHIcrr::neo^+ ^*(Kan^R^)	[16]
LJ231	LJ110 *ΔmanXYZ::cat csc^+^*	K. Jahreis, lab stock
LJB5	LJ231 *ptsG_P384R_ Tn10tet^+^*(Tet^R^)	[46]
LJB17	LJ110 *dgsA*::*cat*	[47]
MG1655	*F^-^, λ^-^, rph-1*	Yale *E.coli* Stock Center
W3110	*F^-^λ^-^ IN(rrnD-rrnE)1 rph-1*	Yale *E.coli* Stock Center
**Bacteriophage**		
P1kc	*lysogen*	[48]

**Table 2:** Plasmids used in this study

Name	Resistance	Properties	Source or Reference
*Escherichia coli* vectors			
pACYC184	Tc^R^		[49]
pACYC184sgrT3HA	Tc^R^	SgrT-3HA (tac_PO_)	this study
pBAD24	Ap^R^		[50]
pBLP2	Ap^R^	EIICB^Glc^-GFP	[51]
pET11a-link-NGFP	Ap^R^		[52]
pET11a-Z-NGFP	Ap^R^	NGFP-Leucinzipper	[52]
pETS	Ap^R^	NGFP-SgrT	this study
pKD3	Ap^R^, Cm^R^		[44]
pKD46	Ap^R^		[44]
pMRB	Kn^R^	EIIB^Glc^-CGFP	this study
pMRBAD-link-CGFP	Kn^R^		[52]
pMRBAD-Z-CGFP	Kn^R^	Leucinzipper-CGFP	[52]
pMRC	Kn^R^	EIIC^Glc^-CGFP	this study
pMRCL	Kn^R^	EIIC^Glc^-linker-CGFP	this study
pMRCL-P384R	Kn^R^	EIIC^Glc^-linker-CGFP-P384R	this study
pMRG	Kn^R^	EIICB^Glc^-CGFP	this study
pMRLB	Kn^R^	Linker-EIIB^Glc^-CGFP	this study
pMRLB-P384R	Kn^R^	Linker-EIIB^Glc^-CGFP-P384R	this study
pRR48	Ap^R^		Parkinson, LKS
pRR48G	Ap^R^	EIICB^Glc^	[53]
pRR48GH	Ap^R^	EIICB^Glc^-5His	[53]
pRR48GH-D388A	Ap^R^	EIICB^Glc^-D388A-5His	this study
pRR48GH-E387A	Ap^R^	EIICB^Glc^-E387A-5His	this study
pRR48GH-G385A	Ap^R^	EIICB^Glc^-G385A-5His	this study
pRR48GH-I296N	Ap^R^	EIICB^Glc^-I296N-5His	this study
pRR48GH-K150E	Ap^R^	EIICB^Glc^-K150E-5His	this study
pRR48GH-K382A	Ap^R^	EIICB^Glc^-K382A-5His	this study
pRR48GH-P384A	Ap^R^	EIICB^Glc^-384A-5His	this study
pRR48GH-P384R	Ap^R^	EIICB^Glc^-384R-5His	this study
pRR48GH-R386A	Ap^R^	EIICB^Glc^-R386A-5His	this study
pRR48GH-T383A	Ap^R^	EIICB^Glc^-T383A-5His	this study
pRR48GH-V12F	Ap^R^	EIICB^Glc^-V12F-5His	this study
pTM30	Ap^R^		[54]
pTM30sgrT	Ap^R^	SgrT	this study
pTM30sgrT3HA	Ap^R^	SgrT-3HA	this study
pTM30sgrTgfp	Ap^R^	SgrT-GFP	this study

*Isolation of chromosomal and plasmid DNA, restriction analysis, PCR and DNA sequencing.* All manipulations of chromosomal or recombinant DNA were carried out using standard procedures as described previously [55]. Plasmid DNA was prepared using QIAprep Spin Miniprep Kit (Qiagen, Hilden, Germany). Restriction enzymes were purchased from New England Biolabs (Schwalbach, Germany) or Fermentas (St. Leon-Rot, Germany) and used according to supplier recommendations. Oligonucleotides for PCR were purchased from Thermo Fisher Scientific (Ulm, Germany). DNA sequencing was commissioned to Scientific Research and Development (Bad Homburg, Germany). Polymerase chain reactions (PCR) were performed as described by [57] using TaKaRa DNA polymerase from Lonza (Köln, Germany).

*Construction of plasmids.* The open reading frame of *sgrT* (*sgrT* ORF) was amplified using chromosomal DNA of LJ110, which is closely related to wild type *E. coli* K-12, with forward primer sgrT+, containing a *Pst*I restriction site and reverse primer sgrT-, which had a *Hind*III restriction site. This PCR product was purified with Wizard DNA purification system (Promega) and cloned into the vector pTM30, resulting in plasmid pTM30sgrT. The expression plasmid pTM30 provides a tac-promoter, an artificial start codon and and an artificial ribosomal binding site as described before [54]. pTM30sgrT3HA carries additional sequences that encode a 3xHA tag (received from pFA6a3HA, Oligonucleotides HA+/-) fused to the C-terminus of SgrT. For pACYC184sgrT3HA the *tac_PO_* and *sgrT3HA* sequence from pTM30sgrT3HA was amplified with oligonucleotides TacPO+ and HA- and cloned into the vector pACYC184. For the bimolecular fluorescence complementation assay the *sgrT* ORF was amplified by PCR (Oligonucleotides pETS+/-) and cloned into the vector pET11a-link-NGFP [52] using the restriction enzymes *Xho*I/*Bam*HI. The genes encoding EIIB^Glc^ (aa 389-477), Linker-EIIB^Glc^ (aa 380-477), Linker-EIIB^Glc^-P384R (aa 380-477), EIIC^Glc^ (aa 1-381), EIIC^Glc^-Linker (aa 1-396), EIIC^Glc^-Linker-P384R (aa 1-396) and EIICB^Glc^ (aa 1-477) were also amplified by PCR and purified. The oligonucleotides were used as follows: EIIB^Glc^ (pMRB+/pMRG-), Linker-EIIB^Glc^ (pMRLB+/pMRG-), Linker-EIIB^Glc^-P384R (pMRLB-P384R+/pMRG-), EIIC^Glc^(pMRG+/pMRC-), EIIC^Glc^-Linker (pMRG+/pMRCL-), EIIC^Glc^-Linker-P384R (pMRG+/pMRCL-P384R-) and EIICB^Glc^ (pMRG+/-). PCR products were cloned into the vector pMRBAD-link-CGFP [52] using the restriction enzymes *Nco*I/*Aat*II or *Sph*I/*Aat*II, respectively. For fluorescence microscopy, an SgrT-GFP fusion protein was created. The *sgrT* ORF was amplified with oligonucleotides SgrT+/SgrT2- and a *gfp* gene was amplified using pBLP2 as template and oligonucleotides Gfp2+/-. Both PCR products were purified and cloned together into pTM30, resulting in pTM30sgrT-gfp. All oligonucleotide sequences used are listed in Table3 in the supplemental materials.

*Site-directed mutagenesis.* We generated defined mutations in the *ptsGHis* gene using the pRR48GH plasmid as a template and the Phusion Site-directed Mutagenesis Kit according to standard protocol (Finnzymes, Vantaa, Finland). The oligonucleotides used are listed in Table 3 in the supplemental materials (K150E+/-, V12F+/-, K382A+/ktpg-, T383A+/ktpg-, P384A+/ktpg-, P384R+/ktpg-, G385A+/ktpg-, R386A+/red-, E387A+/red-,D388A+/red-).

*Construction of strains. JKA1**:*** In order to characterize the consequence of a *sgrRST*-deletion we disrupted the *sgrRST*-wildtype genes via λ Red recombination as described in the protocol of Datsenko and Wanner [44]. Briefly, a chloramphenicol resistance selection marker with flanking regions that are homologous to chromosomal sequences at the 5’ and 3’ end of the *sgrRST* gene region was amplified from template pKD3 [44] by using the primer pair SgrR+ and SgrS-. The PCR product was purified with the Wizard DNA purification system (Promega), treated with *Dpn*I and further enriched by ethanol precipitation. Subsequently, the DNA-fragment was integrated into the chromosome of BW25113/pKD46 [44] via λ Red recombination, resulting in BW25113Δ*sgrRST*::*cat*. JKA1 was created by the transfer of the deletion cassette of BW25113Δ*sgrRST*::*cat* into LJ110 via P1-transduction. PCR of flanking regions was used to confirm the correct integration of the desired gene disruption.

*JKA12*: For crosslinking analysis a strain with a deletion of *ptsG* and *sgrRST* gene regions was created. The *sgrRST* gene region in BW25113/pKD46 was disrupted with a kanamycin resistance cassette via λ Red recombination, using the protocol of Datsenko and Wanner [44]. The deletion cassette of BW25113*ΔsgrRST*::*kan* was then inserted via P1-transduction into LJ120, resulting in JKA12. PCR was used to confirm correct integration of the desired gene disruption.

*JKA17***:** To characterize the interaction of SgrT and EIICB^Glc^ with bimolecular fluorescence complementation a BL21 (λDE3) strain with a *ptsG*-deletion was created via P1-transduction (Δ*ptsG*::*cat* from LJ120). PCR was used to confirm correct integration of the desired gene disruption.

*JKA18*: For fluorescence microscopy one strain with a deletion of *sgrRST* gene region and a chromosomal point mutation in *ptsG* was constructed. The deletion cassette of BW25113*ΔsgrRST*::*kan* was inserted via P1-transduction into LJB5, resulting in JKA18. PCR was used to confirm correct integration of the desired gene disruption. The oligonucleotides are listed in Table 3 in the supplemental materials.

*Western blot analyses*. For Western blot analysis, bacterial cells were treated as described in the section “crosslinking with paraformaldehyde” or grown overnight in LB_0_ with ampicillin and inoculated in fresh medium to an OD_650_ = 0.1. The cultures were grown to early-log phase (OD_650_ = 0.2) and induced with IPTG. Cells were harvested at an optical density at 650 nm of 1 by centrifugation and resuspended in 100 µL sterile water and 100 µL of SDS-PAGE loading buffer (125 mM Tris-HCl (pH 6.8), 2% sodium dodecyl sulphate (SDS), 10% glycerol, 5% ß-mercaptoethanol, 0.01% bromophenol blue). If not described otherwise, samples were heated at 95 °C for 10 min. 15 µL of total cellular proteins were separated by electrophoresis on 0.1% SDS-containing 15% polyacrylamide gels and transferred to a Nitrocellulose membrane (Schleicher & Schuell, Dassel, Germany). For the detection of EIICB^Glc^-His protein derivatives, we used a Penta-His antibody (Qiagen, Hilden, Germany). SgrTec3HA was detected with HA-antibody (kindly provided by Anja Lorberg, University of Osnabrück). Detection of antibody binding was performed using infrared-labeled second antibodies (LI-COR Biosciences, Bad Homburg, Germany).Visualization and quantification were done using an Odyssey infrared imager (LI-COR Biosciences, USA) and the software provided by the supplier (Odyssey 2.1).

*Crosslinking with paraformaldehyde*. For crosslinking of proteins with paraformaldehyde the general procedure from [30] was followed. Cells were grown overnight in LB_0_ media with ampicillin and tetracycline and inoculated in 200 mL fresh medium to an OD_650_ = 0.1. The cultures were grown for one hour at 37 °C and induced with 1mM IPTG. After one hour 0.2% glucose was added to cultures when indicated and cultures were incubated for another hour. Then paraformaldehyde solution (4% in PBS (136 mM NaCl, 2.7 mM KCl, 1.8 mM KH_2_PO_4_, 10 mM Na_2_HPO_4_) was added in a concentration of 0.3%. Cultures were incubated for 20 min at 37 °C while shaking and cells were harvested via centrifugation. The pellet was washed in a lysis buffer (50 mM NaH_2_PO_4_, 300 mM NaCl, 10 mM Imidazol, pH 8.0) and finally resuspended in 5 mL of lysis buffer. 1mM AEBSF was added and cells were disrupted by sonification. Cell debris was removed via centrifugation and the supernatant was used for solubilization of membrane proteins. Therefore 2% triton X-100 was added to the supernatant and incubated at room temperature (RT) for 30 min while mixing. Membranes were removed via ultracentrifugation. The supernatant was then used for protein purification with Ni-NTA Agarose (Qiagen, Hilden, Germany). 1.25 mL Ni-NTA agarose was mixed with 5 mL protein suspension and incubated for one hour at RT. Supernatant was removed via centrifugation and unbound protein was removed using wash buffer (50 mM NaH_2_PO_4_, 300 mM NaCl, 20 mM Imidazol, pH 8.0) twice. 625 µL (1/8) Elution buffer (50 mM NaH_2_PO_4_, 300 mM NaCl, 250 mM Imidazol, pH 8.0) was used to elute purified protein. The same amount SDS sample buffer was added, proteins heated to 95 °C for 10 min to destroy protein complexes, and equal amounts of proteins were analyzed with Western blot analysis. 

*Bimolecular fluorescence complementation.* For bimolecular fluorescence complementation strain JKA17 was used. Protocol and plasmids were used as described in [31,52]. The cells were inoculated in rich medium with 100 µM IPTG, 0.4% arabinose and 0.2% glucose and incubated for three days at 25 °C while shaking. Cells were harvested via centrifugation and resuspended in 1 mL of lysis buffer [52]. OD_420_ was determined and equal amounts of cells used for measuring fluorescence activity in a fluorimeter (Fluorometer Fluostar Optima, BMG LABTECH GmbH, Ortenberg, Germany).

*Fluorescence microscopy.* For standard microscope examination, cells were grown to early logarithmic phase in minimal medium complemented with 0.2% glucose. After 2 hours, 5 µM IPTG or 100 mM arabinose was added, incubated for two hours and cells were fixed on a slide with polylysin. The setup used for fluorescence microscopy consisted of a Zeiss Axioplan2e (Carl Zeiss, Jena, Germany) equipped with a 100× alpha-Plan Fluar objective (NA 1.45) and differential interference contrast (DIC). Images were acquired using a Photometrics CoolSNAP HQ Camera (Roper ScientiWc, Tucson, USA). Fluorescence was excited with a helium lamp and appropriate filter sets were used to adjust excitation and emission wavelengths. The setup was controlled by the Metamorphs v6.2 program (Universal Imaging Corporation, Downingtown, USA). Bright field images were acquired as single planes using t DIC. All fluorescence images were taken from single focal planes and scaled using Metamorphs scale image command. All GFP fusions were taken with 1 sec acquisition time. From all cultures, at least 100 cells were controlled. For unspecific cell wall staining, the cells were incubated with 4µM FM4-64 for 10 min at RT.

## 4. Conclusions

*E. coli* tends to produce acetate during high cell density fermentation. Acetate production takes place when the rates of carbohydrate transport and glycolysis exceed a critical value. Many attempts have been performed to couple carbohydrate uptake rates to metabolic flux in order to avoid overflow mechanisms. The *sgrRST* system provides new regulatory tools to artificially modify glucose uptake rates according to biotechnological needs. Clearly, further fundamental research efforts are necessary to adapt and optimize the *sgrRST* system as an instrument for fine-tuning carbohydrate uptake in biotechnological applications.

## Supplementary Files

Supplementary File 1 Supplementary Material (PDF, 58 KB)
