# Supplementary material for: Characterization of the Interaction Between the Small Regulatory Peptide SgrT and the EIICBGlc of the Glucose-Phosphotransferase System of E. coli K-12
Source: Metabolites. 2012 Oct 16;2(4):756–74. doi: 10.3390/metabo2040756 (PMC3901232; doi:10.3390/metabo2040756)
Supplement: Supplementary File 1 — Supplementary Material (PDF, 58 KB) [file metabolites-02-00756-s001.pdf]

Supplemental material Table 3. Oligonucleotides used in this study.

Name	Sequence (5' – 3')
D388A+	AAC GCC GGG TCG TGA AGC CGC GAC TGA AG
E387A+	AAC GCC GGG TCG TGC AGA CGC GAC TGA AG
G385A+	TGG ATC TGA AAA CGC CGG CTC GTG AAG ACG
Gfp2-	CGC GAT ATC TTT GTA GAG CTC ATC CAT GCC
Gfp2+	GAT GGT ACC GCC GCG CCA GCA CCC G
HA-	CGG GTA CCA GCG TAA TCT GGA ACG
HA+	GGG AGG CTT TAC CCA TAC GAT GTT CC
K150E-	CAA GAT ACT CAG GCA GCT TAA TAC GGT AG
K150E+	GCT TCT TTG CCG GTG AAC GCT TTG TGC C
K382A+	TGG ATC TGG CAA CGC CGG GTC GTG AAG ACG
ktpg-	GTG CTT TAA TCA GCA CGC GGA AGA TGG TG
P384A+	TGG ATC TGA AAA CGG CGG GTC GTG AAG ACG
P384R+	TGG ATC TGA AAA CGC GGG GTC GTG AAG ACG
pETS-	GCG GGA TCC ACT TTC AGA ATT GCG G
pETS+	CGC CTC GAG GCA GTT TTA TCA GC
pMRB+	CAT GCA TGC GCG ACT GAA GAT GC
pMRC-	GCG GAC GTC AGA TCC AGT GCT TTA ATC
pMRCL-	GGG GAC GTC GTC GCT TTT GCA TCT TCA GTC
pMRCL-P384R-	GAT GAC GTC GCG TCT TCA CGA CCC CGC GTT TTC
pMRG-	CGC GAC GTC GGG TTA CGG ATG TAC TC
pMRG+	CCC GCA TGC TTT AAG AAT GCA TTT GC
pMRLB+	CAT GCA TGC GGA TCT GAA AAC GCC
pMRLB-P384R+	CAT GCA TGC GGA TCT GAA AAC GCG GGG TCG
R386A+	AAC GCC GGG TGC TGA AGA CGC GAC TGA AG
red-	TTCAGATCCAGTGCTTTAATCAGCACGCG
SgrS-	AGC AGG TAT AAT CTG CTG GCG GGT GAT TTT ACA CCA ATA CTC AGT CAC ACC ATA TGA ATA TCC TCC TTA
SgrT-	AGG CAA GCT TAC TTT CAG AAT TGC GGT
SgrT+	CCC CTG CAG CAG TTT TAT CAG CAC TAT TTT ACC
SgrT2-	CGC GGT ACC AAG CTT ACT TTC
SrgR+	GGT GGC GCA AAC CAC GCT GAT TTA AAA TCG AAC CAG CCG AGG GTA TTC ATG TGT AGG CTG GAG CTG CTT CG
T383A+	TGG ATC TGA AAG CGC CGG GTC GTG AAG ACG
TacPO+	CAC AGT ACT CTA TCG CTA CGT GAC
V12F-	CAA ATG CAT TCT TAA ACT GCA GCA TAT G
V12F+	CTA ACC TGC AAA AGT TCG GTA AAT CGC TG
